# Educational Supervision – Backbone of Training: How Do We Make It Effective?

**DOI:** 10.1192/bjo.2022.107

**Published:** 2022-06-20

**Authors:** Mrunal Bandawar, Zahra Ali, Laura Jayne Carone, Kallol Sain

**Affiliations:** 1 Birmingham and Solihull Mental Health NHS Foundation Trust, Birmingham, United Kingdom.; 2 Institute of Mental Health, University of Birmingham, Birmingham, United Kingdom

## Abstract

**Aims:**

Assess and improve the educational supervision for the core psychiatry trainees (CT) in the west midlands.

**Methods:**

The data were collected from core psychiatry trainees in West Midland (CT1 – 3) through a Microsoft form sent via the Faculty support team and data are collected (June 2021) from CT's perspective. It involved demographics and questions evaluating quantitative and qualitative overview of educational supervision. We used HEE guidelines and RCPsych recommendations. Similarly, we used a modified questionnaire to anonymise educational supervisors’ (ES) perspectives in the West Midlands School of psychiatry annual Education day conference (January 2022).

**Results:**

Trainees Perspective: 40% out of 123 trainees responded, of which 35% were CT1, 40% were CT2, and 25% were CT3. 59% said that CT in psychiatry was their first training job in the UK. In the quantitative overview, 25% of the trainees responded their 1st contact with their ES was more than six weeks after beginning their 1st post, and 29% expressed their 1st meeting more than six weeks following the start of their 1st post in the academic year. 67% met adequate standards in the quantity of educational supervision in an academic year. In qualitative overview, 19% didn't understand the role of ES, and 54% didn't know how to raise concerns about ES. The thematic analysis of the feedback suggested points of improvement as supervisions not being ‘tick-box’ exercises and accessibility of ES.

The trainer's perspective: 60% of attendees responded, 71.4% were ES. All the responding ES answered that they would arrange their 1st meeting six weeks before the start of the academic year. Almost all suggested the most common difficulty in educational supervision as availability of time, considering clinical workload for both ES and CTs. All respondents knew that the number of meetings would be as many as trainees wanted in an ideal/needful situation. From the thematic analysis of free text, almost all responded lack of time was a barrier in providing the supervision reflecting on their ability to engage with the trainees.

**Conclusion:**

Suggested recommendations were to raise awareness among the trainees through workshops at induction to explain the aim and objective of educational supervision and to have a guided list of suggested topics to discuss in supervision. For trainers, further training about HEE & RCPsych guidance about Educational supervision would be helpful. Educational leads need to engage in job planning. A comparison between Trainees and trainers feedback through the GMC survey may help to compare with the national picture.

